# Entropy Generation Analysis in Turbulent Reacting Flows and Near Wall: A Review

**DOI:** 10.3390/e24081099

**Published:** 2022-08-10

**Authors:** Amsini Sadiki, Senda Agrebi, Florian Ries

**Affiliations:** 1 Institute of Reactive Flows and Diagnostics, Technical University of Darmstadt, 64287 Darmstadt, Germany; 2 Laboratoire de Modélisation Mécanique, Energétique et Matériaux, ISTA-Kinshasa, Avenue Aérodrome N° 3930, Commune de Barumbu, Kinshasa BP 6593, Democratic Republic of the Congo; 3 Institute of Energy and Power Plant Technology, Technical University of Darmstadt, 64287 Darmstadt, Germany; 4 Mechanics, Modelling Energy and Materials Unit (M2EM), National School of Engineers of Gabes, Gabes 6029, Tunisia

**Keywords:** review, entropy generation, exergy, numerical modeling approaches, combustion systems, near wall, applications, optimization

## Abstract

This paper provides a review of different contributions dedicated thus far to entropy generation analysis (EGA) in turbulent combustion systems. We account for various parametric studies that include wall boundedness, flow operating conditions, combustion regimes, fuels/alternative fuels and application geometries. Special attention is paid to experimental and numerical modeling works along with selected applications. First, the difficulties of performing comprehensive experiments that may support the understanding of entropy generation phenomena are outlined. Together with practical applications, the lumped approach to calculate the total entropy generation rate is presented. Apart from direct numerical simulation, numerical modeling approaches are described within the continuum formulation in the framework of non-equilibrium thermodynamics. Considering the entropy transport equations in both Reynolds-averaged Navier–Stokes and large eddy simulation modeling, different modeling degrees of the entropy production terms are presented and discussed. Finally, exemplary investigations and validation cases going from generic or/and canonical configurations to practical configurations, such as internal combustion engines, gas turbines and power plants, are reported. Thereby, the areas for future research in the development of EGA for enabling efficient combustion systems are highlighted. Since EGA is known as a promising tool for optimization of combustion systems, this aspect is highlighted in this work.

## 1. Introduction

In many engineering applications, especially in a variety of technology sectors and energy conversion systems, there is an increasing need for more energy-efficient thermofluid components and devices driven by both environmental sustainability and efficient operating condition demands. In this respect, it is now recognized that an analysis of local entropy generation consisting of minimizing the irreversibilities in the transport processes and physico-chemical events can be used as an effective means to optimize the performance of current and future thermofluid-based applications and to support novel developments.

The objectives of an entropy generation analysis are generally threefold. First, this requires the formulation of appropriate models that describe the evolving transport processes, including the entropy transport equation, considering the finite size of actual systems and the finite speeds of ongoing real processes. Second, this not only allows the identification of the causes of inefficiency of processes but also permits the evaluation of the significance (location and magnitude) of irreversibilities generated by each specific transport process. Third, this aids to delimit the evolution of the processes and, at the same time, gives access to the control and possible minimization of irreversibilities.

**Figure 1:**
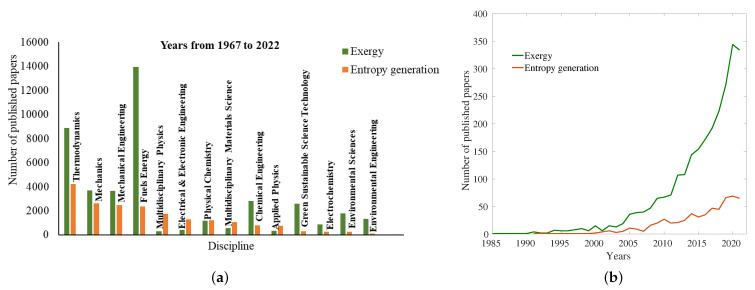
The number of published papers on exergy and entropy generation (**a**) as a function of discipline from 1967 to 2022 (**b**) in combustion systems from 1985 to 2021 (data extracted from [35]).

The second and third aspects are essentially addressed in this review due to the strong pressure of the search for more efficient operating conditions, which is continuously increasing. In this respect, the second law of thermodynamics and the entropy production analysis in particular have been widely used to assess the sources of irreversibility in components and systems. Figure 1 shows the relevance of the field in different technical, industrial and environmental areas and, at the same time, gives inference on the future scope of this field in various disciplines and specifically in the field of combustion.

All this is demonstrated by the number of published papers devoted to entropy generation and exergy in different disciplines since 1967 (Figure 1a) and specifically by the increased evolution of published papers dedicated to the field in combustion systems since 1985 (Figure 1b) thermal devices in which viscous and thermal effects for convective heat transfer [1], as well as combined mass and heat transfer phenomena [1], play a significant role.

Thereby, the majority of the reported studies were restricted to a global analysis. The assessment of local sources of irreversibilities, i.e., local entropy production, was often based on empirical correlations for pressure drop and heat transfer performance. In previous reviews, fundamentals of second-law-based analysis [1,2,3,4,5] and related applications have been reported. These comprised system-level analysis, commonly called exergy analysis, to calculate the net rate of energy degradation.

From an engineering perspective, this concept of entropy generation minimization can be therefore useful as a design tool in order to avoid the imminent loss of available mechanical power in thermo-fluid systems [6,7]. A detailed description of the theoretical background of such an entropy generation analysis has been provided in [5,8].

Regarding the entropy generation analysis based on compuational fluid dynamics (CFD), commonly, the local form of the second law is applied to consider the thermodynamic irreversibilities. This allows evaluation of the total entropy generation of a system and also to explore how irreversibilities are allocated within the system [5]. According to the minimal entropy generation approach and CFD, irreversibility sources have been investigated for a wide range of thermo-fluid processes, including laminar and turbulent flow regimes in wall-bounded flows [9,10,11,12,13,14], supercritical flows [15,16,17], reacting flows [18,19,20,21,22] and impinging flows featuring complex heat transfer processes [23,24,25]. The theory and applications of entropy generation concept based on CFD have been reported for different types of engineering systems in [4,5,26].

Concerning the entropy generation analysis in turbulent heat and fluid flows, only a few direct numerical simulation (DNS) investigations are found in the literature. Okong’o and Bellan [16] used DNS to analyze the entropy production in supercritical transitional mixing layers. They stated that entropy generation is promising for the description of the behavior of small-scale turbulent motions.

Farran and Chakraborty [20] (see also [27]) conducted DNS of a freely propagating premixed flame. They evaluated the turbulent second law efficiency and compared with that of laminar flames. Ries et al. [15,23] provided two comprehensive DNS databases of entropy production rates of a supercritical injection process and an inclined impinging jet configuration, and these appear useful for model development and validation in the context of large eddy simulation (LES) and Reynolds-averaged Navier–Stokes (RANS) framework, respectively.

Contrary to DNS, investigations on entropy generation analysis within the RANS approach have been reported in many numerical studies. It is essential to mention that most of these studies are either experiments supported by RANS-based simulations or numerical mainly using RANS-based models. Mohseni and Bazargan [17] analyzed the impact of the wall heat flux variation in a heated vertical tube at supercritical conditions. They found that the efficiency of the system rises near the pseudo-boiling point operating conditions.

Shuja et al. [24] used the minimum entropy generation concept to analyze the local entropy production in an impinging jet while evaluating the prediction capability of different turbulence models. Kock and Herwig [13,28] developed wall-functions for modeling the entropy generation rates near the wall and applied the resulting model to assess the heat transfer efficiency of turbulent shear flows. Several other RANS investigations are found in the literature that present various studies aiming at performance or design optimization of thermodynamic systems using CFD-based local entropy production techniques, e.g., [20,29,30].

Within the LES context, entropy generation analysis is scarcely addressed in the literature, largely due to the complexity in modeling the unclosed subgrid irreversibility contributions. In addition to Ries et al. [31], who focused on non-reacting flows, a review on reacting flows up to 2014 was provided by Safari et al. [32], while Agrebi et al. [33] reviewed the recent LES-based investigations on the analysis of entropy generation applied to combustion systems up to 2020.

This paper will not explore the effect of the chemical kinetics and thermal diffusion models on the differential entropy inequality (DEI)—the local form of the second law of thermodynamics. This analysis was done elsewhere. Some reduced chemical kinetics mechanisms are used, which produce violations of the differential entropy inequality as reported by Ream et al. [34].

The present paper focuses on reviewing different contributions devoted thus far to entropy generation analysis in turbulent combustion systems. Special attention is paid to experimental and numerical modeling works along with selected applications of practical interest. On the one hand, the difficulties of performing comprehensive experiments that support the understanding of entropy generation phenomena will be outlined.

On the other hand, the modeling of the exergy balance together with the entropy production for reacting flows under turbulent operating conditions will be presented. First, the governing equations will be introduced as they are needed to describe such processes in turbulent flows. Apart from DNS, the description in both the RANS and LES contexts with focus on the transport equation of entropy will be outlined followed by the formulation, which includes the turbulence/chemistry interactions. Thereafter, the required RANS and LES models and associated closures for the filtered entropy equations will be provided along with different modeling degrees of the entropy production terms.

Some applications will be shown and discussed within each modeling approach. The survey will specially account for various parametric studies reported in the literature, including wall boundedness conditions, flow operating conditions, combustion regimes and fuels along with alternative fuels and configuration geometries. Since the EGA is known as a promising tool for optimization of combustion systems, this aspect will be highlighted throughout the paper. The last section will be devoted to concluding remarks and future research aspects.

## 2. Experimental Investigations and 1D Calculations of Exergy and Energy

Turbulent combustion systems have been investigated using modern experimental techniques since the 1990s (see in [36]). However, it turns out that existing experimental data do not provide entropy production rate in such systems. This section reviews the main contributions dealing with the entropy generation analysis in which a 1D modeling approach of the exergy balance is used as usually done in complex configurations of industrial interest.

Some of them rely on experimental data gained from very few available experimental investigations. Most of the experimental studies [37,38,39,40,41,42,43,44,45,46] dealing with the evaluation of combustion systems irreversibilities are found for internal combustion engines (IC engines), gas turbines, burners and power plants. Therefore, the modeling of exergy and energy balance is first introduced, followed by selected applications in the area of IC engines, gas turbines and power plants.

### 2.1. Modeling of Exergy and Energy Balance

Recently, the application of the entropy generation analysis has been extended to practical energy conversion systems, especially where complex phenomena together with combustion or chemical reactions usually take place. The analysis of the entropy generation in combustion systems, such as gas turbine combustion chambers, has been one of the most interesting issues in the recent years, since there are numerous irreversible processes in the gas turbine combustors that result in the loss of exergy.

The earlier works stem from Teng et al. [47], who derived the entropy transport equation to determine the rate of local entropy generation in multicomponent laminar reacting flows. Nishida [2] focused specially on laminar diffusion flames. See also Datta and Som [48] who considered energy and exergy balance in a gas turbine combustor. In a combustion system, the exergy balance for a control volume can be given as [49,50]:

(1) $$\sum \dot{E}x_{in}=\dot{E}x_{work}+\dot{E}x_{dest}+\dot{E}x_{heat}+\dot{E}x_{exhaust}$$

where $\dot{E}x_{in}$, $\dot{E}x_{work}$, $\dot{E}x_{dest}$, $\dot{E}x_{heat}$ and $\dot{E}x_{exhaust}$ are the input exergy, the output exergy, the destroyed exergy during combustion, the exergy of heat losses and the exergy of the exhaust gases, respectively. The exergy input is the exergy of the air and fuel at the inlets expressed as [49,50]:

(2) $$\dot{E}x_{in}=\dot{E}x_{air}+\dot{E}x_{fuel}$$

(3) $$\dot{E}x_{air}={\dot{m}}_{air}\left[C_{p,air}\left(T_{air}-T_{0}\right)-T_{0}\ln\left(\frac{T_{air}}{T_{0}}\right)+RT_{0}\ln\left(\frac{P_{0}}{P_{air}}\right)\right]$$

(4) $$\dot{E}x_{fuel}={\dot{m}}_{fuel}\left[1.0401+0.1728\frac{H}{C}+0.0432\frac{O}{C}+0.2169\frac{S}{C}\left(1-2.0628\frac{H}{C}\right)\right]LHV$$

In Equation (3), R is the gas constant and ${\dot{m}}_{air}$, $T_{air}$, $C_{p,air}$ and $P_{air}$ express the mass flow, temperature, specific heat capacity and pressure of the air at the inlets. For the fuel exergy given by Equation (4), ${\dot{m}}_{fuel}$ and *LHV* are the mass flow inlet and lower heat value of the fuel, however *H*, *C*, *O* and *S* stand for the mass fractions of hydrogen, carbon, oxygen and sulfur contents of fuel, respectively.

The exergy of useful work called also the exergy output, where $\dot{E}x_{work}$ is exergy associated with work done by the system in main chamber. For IC engines, this can be defined as [49,50]:

(5) $$\dot{E}x_{work}=\Omega \times Tr$$

where Ω and *Tr* are the angular speed and the engine torque, respectively. Another way to define the work exergy in IC engines is relying on the crank angle Θ, cylinder pressure $P_{cylinder}$, and volume *V*, as follows [49,50]:

(6) $$\frac{d\dot{E}x_{work}}{d\Theta }=\left(P_{cylinder}-P_{0}\right)\frac{dV}{d\Theta }$$

The exergy associated with heat transfer losses across boundaries of the investigated system, $\dot{E}x_{heat}$ is exergy associated with heat transfer across boundary, $Q_{loss}$, which is calculated as [49,50]:

(7) $$\dot{E}x_{heat}=\left(1-\frac{T_{0}}{T}\right){\dot{Q}}_{loss}$$

The exergy related to the exhaust gases released after the combustion at mass flow ${\dot{m}}_{exhaust}$ through the exhaust manifold involves two different components—namely, the physical exhaust exergy $\dot{E}x_{exhaust,ph}$ and the chemical exhaust exergy ${\dot{Ex}}_{exhaust,ch}$ [49,50]:

(8) $$\dot{E}x_{exhaust}=\dot{E}x_{exhaust,ph}+\dot{E}x_{exhaust,ch}$$

The physical exhaust exergy $\dot{E}x_{exhaust,ph}$, known also as the thermo-mechanical exergy, is expressed as a function of the exhaust gases pressure, $P_{exhaust}$, and temperature, $T_{exhaust}$, which is much higher than that of atmospheric temperature ($T_{0}$) [51] is given by [49,50]:

(9) $$\dot{E}x_{exhaust,ph}={\dot{Q}}_{exhaust}+{\dot{m}}_{exhaust}T_{0}\left[C_{p,exhaust}\ln\left(\frac{T_{0}}{T_{exhaust}}\right)-R_{exhaust}\ln\left(\frac{P_{0}}{P_{exhaust}}\right)\right],$$

where $Q_{exhaust}$ is the heat energy taken by exhaust gases and defined as a function of the mass flow rate, specific heat and temperature of the exhaust gases as:

(10) $${\dot{Q}}_{exhaust}={\dot{m}}_{exhaust}C_{p,exhaust}\left(T_{exhaust}-T_{0}\right).$$

The last term of the exhaust exergy, which is the chemical exhaust exergy, $\dot{E}x_{exhaust,ch}$, is expressed as [49,50,52,53,54]:

(11) $$\dot{E}x_{exhaust,ch}={\dot{m}}_{exhaust}RT_{0}\sum \ln\frac{Y_{\alpha }}{Y_{\alpha }{}_{0}}$$

where $Y_{\alpha }$ is the molar fraction of the *i*th species in the exhaust gas and $Y_{ir}$ is the molar fraction of the same ith species in the reference environment.

The remaining term from the exergy balance, which is the destroyed exergy during combustion, $\dot{E}x_{dest}$ is estimated through entropy generation by:

(12) $$\dot{E}x_{dest}=T_{0}{\dot{S}}_{gen}$$

However, experimentally, it is difficult to calculate ${\dot{S}}_{gen}$ since it requires the gradients of velocity, temperature and species concentrations, which is complicated despite the advanced measure techniques. Thus, the exergy destroyed during combustion is obtained through subtracting $\dot{E}x_{work}$, $\dot{E}x_{heat}$ and $\dot{E}x_{exhaust}$ from the exergy input, $\dot{E}x_{in}$.

Finally, the exergy efficiency of the investigated system can be obtained from the ratio of exergy output (exergy of work) and the input exergy (alternatively from the ratio of total exergy losses and the input exergy) as follows:

(13) $$\eta_{ex}=\frac{\dot{E}x_{work}}{\dot{E}x_{in}}=1-\frac{\dot{E}x_{loss,total}}{\dot{E}x_{in}}=1-\frac{\dot{E}x_{dest}+\dot{E}x_{heat}+\dot{E}x_{exhaust}}{\dot{E}x_{in}}.$$

On reciprocating internal combustion (IC), in addition to the two major types—namely, spark-ignition (SI) or compression-ignition (CI) engines—hybrid engines are gaining in importance in addition to fully electric concepts. The performance of such engines is affected by various operating parameters at different load and speed conditions. Some studies have suggested improving engine performance by varying designed operating parameters, such as speed, combustion chamber geometry, fuel injection pressure, fuel injection timing and others. Another method of improving performance can be through the identification and minimization of engine energy losses. To understand the behavior and the complete performance of IC engines, it is necessary to perform the thermodynamic analysis of the real engine operation.

In the following, some contributions are presented, and recent reviews are mentioned. In all cases, the 1D simulations are applied, from which the major reasons of entropy production appeared to be the heat conduction, the viscous dissipation, the mass diffusion and the chemical reaction.

### 2.2. Applications in Internal Combustion (IC) Engines

Dealing with IC engines, reviews have been produced in [5,55,56]. Rakopoulos et al. [55] reported on the identification and quantification of second-law efficiencies and the irreversibilities of various processes and subsystems in IC engines. A detailed reference concerning the most commonly used types of internal combustion engines, i.e., spark ignition, compression ignition (direct or indirect injection), turbocharged or naturally aspirated, during steady-state and transient operation.

All of the subsystems (compressor, aftercooler, inlet manifold, cylinder, exhaust manifold and turbine) were also accounted for. Veena Chaudhary et al. [56] provided a comprehensive review that considered studies from 1957 to 2015. They presented the main objectives of the application of exergy analysis to internal combustion engines (1) to weigh the various processes and devices calculating the ability of each one of these to produce work, (2) to identify the processes that lead to exergy destruction, (3) to quantify the various losses and destruction, (4) to analyze the effect of various design and thermodynamic parameters on exergy destruction and losses, (5) to propose measures for minimization of destruction and losses, to increase the overall efficiency, and (6) to analyze the effect of various thermodynamic parameters and engine design parameters on exergy destruction.

In this respect, Ghazikhani et al. [38] conducted an experimental study on an indirect injection diesel engine to investigate the impact of Exhaust Gas Recirculation (EGR) temperature on irreversibilty and brake specific fuel consumption. Under variable operating engine speeds, they found that about 60% to 70% of the input exergy is destroyed by irreversibilities. The increase of EGR temperature reduces the amount of destroyed exergy.

The effect of injection pressure in a four stroke four-cylinder direct injection diesel CI engine was investigated by Özkan et al. [42]. Their results showed that the exergetic efficiency is inversely proportional to the injection pressure since the power required for injection pump to pressurize the fuel is greater than the increased engine power associated with the injection pressure increase. However, it remains possible to increase exergetic efficiency of a CI engine by adjusting the injection pressure.

Focusing on the effect of fuels on the exergy efficiency of IC engines, several studies [37,40,41,43,44,46] (see also in [5]) have been performed using alternative fuels and biofuels.

Canakci et al. [37] performed energy and exergy analysis of a four cylinder, turbocharged diesel engine fueled with various biodiesels, which are the soy bean oil methyl ester (SME) and yellow grease methyl ester (YGME). They reported that biodiesels provide similar fuel exergy input, exergetic efficiency, exergy destruction and losses to diesel.

Further investigations on second law analysis of biofuels in IC engines were conducted by Sekmen et al. [39] where the petroleum diesel fuel and biodiesel soya bean oil methyl fuels were tested in four-cylinder, direct injection diesel engines. The obtained results supported those found in [37], where it is stated that the exergetic efficiency values were similar for both fuels.

**Figure 2:**
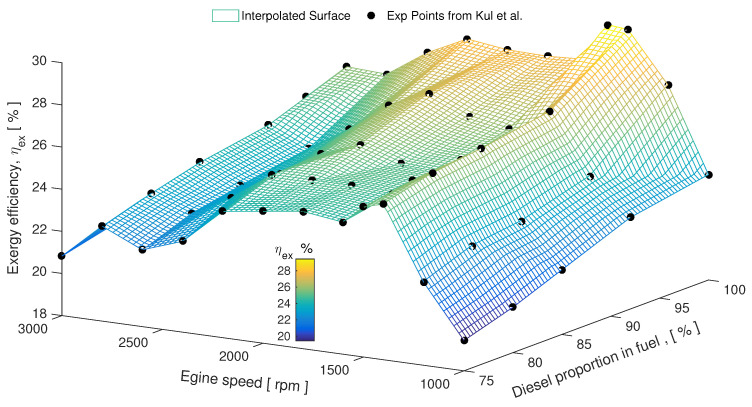
Variation of the exergy efficiency with the engine speed for different fuel blends prepared with a mixture of biodiesel and diesel in different proportions (modified from Kul et al. [43] experiment).

Other investigations on biodiesel were performed by Kul et al. [43] in a single cylinder, water-cooled diesel engine fueled by biodiesel, diesel and 5% bioethanol blends at different engine speeds between 1000 and 3000 rev/min. It was found, as depicted Figure 2, that pure diesel fuel (diesel proportion in fuel blend id 100%) had a slightly higher exergetic efficiency than other fuel blends, and all the results were quite close to each other. The maximum exergy efficiency for all fuel blends were obtained for 1400 rev/min.

**Figure 3:**
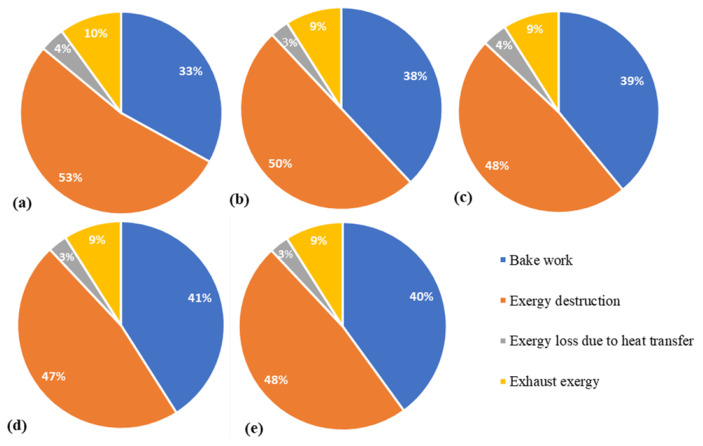
Exergy distribution of different tested fuels. (**a**) Pure diesel. (**b**) Sunflower biodiesel blends. (**c**) Corn biodiesel blends. (**d**) Canola biodiesel blends. (**e**) Restaurant waste biodiesel blends (modified from [44]).

Recently, other biodiesel fuels extracted from different oil-basis materials were evaluated by Jannatkhah et al. [44] in a direct injection and four-cylinder diesel engine in terms of exergy efficiency. The biodiesel fuels investigated were extracted from sunflower oil, corn oil, canola oil and restaurant waste oil. As shown in Figure 3, a decrease of exergy losses was detected with the use of biodiesel, and the Canola biodiesel blends provided the highest exergy efficiency.

### 2.3. Applications in Gas Turbine and Power Plants

Investigations dealing with experimental investigations based on second law in gas turbines and power plants are rarely reported [57,58,59,60,61,62]. Regarding gas turbines, Ibrahim et al. [57] conducted energy and exergy analysis of gas turbine power plants, including the compressor, combustion chamber and turbine.

All the calculations relied on experimental data gained from measurements taken at the power plant—namely, the ambient temperature, air mass flow rate, compressor outlet pressure, compressor pressure ratio, compressor inlet pressure, compressor oulet temperature, gas turbine outlet and inlet temperatures—while estimating the exergy efficiency through the inlet and outlet exergy for each component, they found that the largest amount of irreversibilities occurs in the combustion chamber (about 61.8% of exergy efficiency less than the compressor with 92% and the turbine with 82%).

They proposed measures for reducing energy and exergy losses through the system and for improving the overall system efficiency. Researcher attention has been increasingly given to the combustion chamber in terms of exergy analysis. In this respect, Ismail et al. [62] tested a double-layer micro porous media burner for premixed butane-air combustion with different mixture fractions ranging from lean to rich fuel mixtures (equivalence ratios from Φ = 0.6 to Φ = 1.2) and quantified the entropy generation. The maximum thermal and exergy efficiencies were achieved for Φ = 1.2 and the lowest for Φ = 0.8. Moreover, the lowest values of entropy production, the energy loss and the exergy destroyed were detected for Φ = 1.

Even though the experiments were not reported, Temraz et al. [63] calculated the overall thermal efficiency and the exergetic efficiency of each component in a complex application, the solar-assisted combined cycle power plants (CCPPs) for different solar field capacities. They relied on the experimental data taken from the plant. Further contributions in this respect can be found in [64,65].

Previously, Aljundi [66] performed an exergy and energy analysis in thermal power plant in which each and every component was analyzed separately, and an energy and exergy loss in each component was quantified. The identification was done for the component with the largest energy loss and exergy destruction. The largest energy loss was found to be the condenser where 134 MW is lost to the environment and only 13 MW is lost in the boiler system.

On the other hand, the maximum exergy destruction was found to be in the boiler, which also featured the maximum percentage ratio of exergy destruction of about 77% of the total exergy destruction in the plant. Furthermore, the major source of irreversibility in the boiler was the chemical reaction in combustion chamber. In order to reduce the exergy destruction in the combustion chamber, preheating of combustion air was done and air fuel ratio reduced. Such an outcome was also monitored by Vuckovic [67].

Dang et al. [68] analyzed the energy and exergy of thermal power plant at design and off design load, in particular at two different loads, i.e., 100% and 70% loads. The energy and exergy at inlet and outlet point of each component was calculated and specified with the help of data taken from the plant. A review by Umesh et al. [69] provides detailed report on various studies on thermal power plants up to 2018 while throwing light on the scope for further research.

A recent review was reported by Muhammad et al. [70]. Further energy and exergy analysis of stationary, aircraft gas turbines are reported in [71,72,73,74,75,76,77,78,79] and summarized in Table A1 (see Appendix A) where the most recent and relevant works related to the entropy generation analysis in various applications and accounting for the associated approaches (numerical modeling and/or experimental) are presented.

### 2.4. Intermediate Conclusions

In both applications, it appeared clearly that only investigations limited to exergy balance aiming at identifying the exergy efficiency are generally performed without paying attention to 2D or/and 3D distribution map of each exergy contribution term throughout the turbulent reacting flow system under study. For practical systems, the 1D calculations led generally to non convincing outcome, simply since the sources of irreversibility in reacting flows are in general directly driven by the viscous flow and the temperature dissipation rate as well as the level of heat released by the chemical reactions.

Even though such quantities can be directly linked to pressure loss, heat transfer at walls, fuel calorific value and turbulent mixing and therefore carefully studied and optimized usually during the design of real combustion, they cannot be well captured by 1D simulations. Furthermore, since, inside the combustion chamber, many processes may be involved in exergy destruction, it is difficult with experiments to determine which are the main processes responsible for entropy production.

In fact, the gradients of velocity, temperature and species concentration, which are needed to calculate both viscous and thermal dissipation as well as chemical reaction and mass diffusion contributions in the entropy generation are still challenging even with advanced measurement techniques. Dealing with complex 3D combustion systems, it is thus indispensable to properly monitor the sources of irreversibility by means of numerical models well assessed for design.

## 3. Entropy Generation Analysis Using DNS

Numerous numerical studies have been performed on entropy generation in non-reactive flows involving flow and heat transfer [1,15,24,31,80,81,82,83,84], combined heat and mass transfer [85,86], mixed convection and radiation [87,88] and fuel cells [89,90]. Subsequently, the entropy analysis was extended to reactive flows in combustion systems, where the viscous dissipation, heat transfer and mass diffusion entropy source terms are simultaneously at play with an additional contribution due to chemical reaction. Therefore, DNS-based analyses are scarcely found in the literature.

This section outlines all the classical governing equations needed to describe a reacting flow (balance equations of mass, momentum, energy and species) together with models of entropy production in DNS context. Some applications will complete this overview.

### 3.1. Governing Equations and DNS

The entropy generation analysis of turbulent combustion processes based on direct numerical simulations (DNS) has been recently enabled with the growth of the high-performance computing. However, this analysis is rarely found in the literature [20,27] and first requires the solution of the transport equations that govern the reacting fluid flow—namely, the mass, momentum, energy and species equations:

(14) $$\frac{\partial \rho }{\partial t}+\nabla .\left(\rho \vec{u}\right)=0$$

(15) $$\frac{\partial \left(\rho \vec{u}\right)}{\partial t}+\nabla .\left(\rho \vec{u}\vec{u}\right)=\nabla .\overset{=}{\sigma }+\rho \vec{F}$$

(16) $$\frac{\partial \left(\rho e\right)}{\partial t}+\nabla .\left(\vec{u}\left(\rho e+P\right)\right)=\nabla .\left(\lambda_{eff}T-\sum_{\alpha =1}^{N_{s}}h_{\alpha }{\vec{J}}_{\alpha }+\left(\overset{=}{\tau }.\vec{u}\right)\right)+S_{h}$$

(17) $$\frac{\partial \left(\rho Y_{\alpha }\right)}{\partial t}+\nabla .\left(\rho Y_{\alpha }\vec{u}\right)=-\nabla .{\vec{J}}_{\alpha }+r_{\alpha }M_{\alpha },\;\;\alpha =1,\ldots ,N_{s}-1.$$

In Equation (14), ρ is the fluid density and $\vec{u}$ is the velocity vector. In Equation (15), the quantity $\overset{=}{\sigma }$ and $\vec{F}$ stand for the stress tensor and the externally applied body forces, respectively. Body forces can be applied to individual species if desired by multiplying by the respective mass fraction and summing over all the species. The stress tensor is given by:

(18) $$\overset{=}{\sigma }=\overset{=}{\tau }-p\overset{=}{I}\;,\;\overset{=}{\tau }=\mu \left(\nabla \vec{u}+\nabla {\vec{u}}^{T}\right)+\mu_{b}\left(\nabla .\vec{u}\right)\overset{=}{I}.$$

This includes contributions from both the thermodynamic pressure *p* and viscous stress, $\overset{=}{\tau }$. The notation *I* stands for the identity tensor. Here, μ and $\mu_{b}$ are the dynamic and bulk viscosities, respectively. The Stokes hypothesis is used to approximate the bulk viscosity as $\mu_{b}$ = −2/3μ. The quantity *e* in Equation (16) is the the total mass specific energy, $h_{\alpha }$ represents the mass specific enthalpy of species α, $\lambda_{eff}$ is the effective thermal conductivity, ${\overset{=}{\tau }}_{eff}$ is the effective viscous stress tensor, and *T* is the absolute temperature. $J_{\alpha }$ is the diffusive mass flux vector for species α, and *S_h_* is a source term presented by:

(19) $$S_{h}=-\sum_{\alpha =1}^{N_{s}}h_{f,\alpha }^{0}r_{\alpha }$$

where $h0_{f,\alpha }$ and $r_{\alpha }$ are the mole specific enthalpy of formation and the molar net rate of production of species α, respectively. The rate production of each species in the mixture is obtained as follows:

(20) $$\sum_{\alpha =1}^{N_{s}}{\upsilon^{\prime }}_{\alpha ,r}M_{\alpha }\to \sum_{\alpha =1}^{N_{s}}{\upsilon^{\prime \prime }}_{\alpha ,r}M_{\alpha },\;\;\;r=1,\;\ldots ,\;N_{r}$$

(21) $$r_{\alpha ,r}=\left({\upsilon^{\prime \prime }}_{\alpha ,r}-{\upsilon^{\prime }}_{\alpha ,r}\right)k_{r}\prod_{\alpha =1}^{N_{s}}c_{\alpha }^{{\upsilon^{\prime }}_{\alpha ,r}},\;\alpha =1,\;\ldots ,\;N_{\alpha };\;r=1,\;\ldots ,\;N_{r}$$

(22) $$r_{\alpha }=\sum_{r=1}^{N_{r}}r_{\alpha ,r}=\sum_{r=1}^{N_{r}}\left[\left({\upsilon^{\prime \prime }}_{\alpha ,r}-{\upsilon^{\prime }}_{\alpha ,r}\right)k_{r}\prod_{\alpha =1}^{N_{s}}c_{\alpha }^{{\upsilon^{\prime }}_{\alpha ,r}}\right],\;\alpha =1,\;\ldots ,\;N_{s}$$

(23) $$K_{r}=A_{r}T^{\beta_{r}}\exp\left(\frac{-E_{a,r}}{RT}\right)\;,\;\;\;r=1,\;\ldots ,\;N_{r}\;$$

Note that $\upsilon^{\prime }$ and $\upsilon^{\prime \prime }$ are the stoichiometric coefficients on the reactants and products side of the equation; *N_r_* is the total number reactions and *N_s_* the number of species, respectively. $M_{\alpha }$ and $c_{\alpha }$ stand for the the molecular mass and molar concentration of species α. The rate constant of a chemical reaction, *K_r_* is following the Arrhenius law where *A_r_* is the pre-exponential factor, *E_a,r_* is the activation energy, *R* the universal gas constant, and β is a dimensionless number of order 1.

Thus, for each species to be included in the model, an extra species equation is added, up to a total of *N_s_*-1 species equations. Thereby, the density, ρ and velocity vector, $\vec{u}$ are both properties of the bulk flow while the mass fraction, $Y_{\alpha }$, diffusive mass flux vector, $J_{\alpha }$ and net rate of production, $r_{\alpha }$ are all properties of individual species.

Note that the heat and mass transfer processes are modeled by ignoring the Soret and Dufour effects since it is proven from several previous DNS-based computational analysis [91,92,93,94,95,96,97,98,99,100,101,102,103,104,105,106], that this will not cause much loss of generality. In the case of most hydrocarbon-air and hydrogen-air flames, these effects did not have a significant role [107] except for the extremely lean hydrogen-air flames. Following the Safari et al. [32] investigation, which also did not consider the Soret and Dufor effects, the transport equation of the specific entropy is given in a component form by:

(24) $$\begin{array}{cc} \; & \frac{\partial \rho s}{\partial t}+\frac{\partial }{\partial x_{i}}\left(\rho u_{i}s\right)=\frac{\partial }{\partial x_{i}}\left(\rho D_{m}\frac{\partial s}{\partial x_{i}}\right)+\frac{\partial }{\partial x_{i}}\left(\frac{\lambda }{T}\left(1-\frac{1}{Le}\right)\frac{\partial T}{\partial x_{i}}\right)\\ \; & +\underset{\Pi_{v}}{\underset{︸}{\frac{1}{T}\tau_{ij}\frac{\partial u_{i}}{\partial x_{j}}}}+\underset{\Pi_{q}}{\underset{︸}{\frac{\lambda }{T^{2}}\frac{\partial T}{\partial x_{i}}\frac{\partial T}{\partial x_{i}}}}+\underset{\Pi_{d}}{\underset{︸}{\sum_{\alpha =1}^{N_{s}}\rho D_{\alpha }\frac{R_{\alpha }}{X_{\alpha }}\frac{\partial X_{\alpha }}{\partial x_{i}}\frac{\partial Y_{\alpha }}{\partial x_{i}}}}-\underset{\Pi_{ch}}{\underset{︸}{\frac{1}{T}\sum_{\alpha =1}^{N_{s}}\mu_{\alpha }{\dot{\omega }}_{\alpha }}} \end{array}$$

The first two terms on the left hand side of Equation (24) stand for the accumulation and convection processes, respectively. On the right hand side (RHS), the first term expresses the molecular diffusion contributions to the entropy evolution. The second term (on the RHS) is the redistribution of entropy due to the non-unity Lewis number, and its volume integral vanishes according to the divergence theorem; therefore, it is not involved in the entropy generation. The last terms on the RHS of Equation (24) represent the sources of entropy generation in combustion systems.

This is generally related to four different mechanisms: viscous dissipation (Π_*v*_), heat transfer (Π_*q*_), mass diffusion of species (Π_*d*_) and chemical reaction (Π_*ch*_), respectively. These terms appear in an unclosed form and need modeling. Note that λ, *c_p_*, $R_{\alpha }$, $\mu_{\alpha }$, *D_m_* and $D_{\alpha }$ are the thermal conductivity, specific heat capacity, gas constant of species, specific chemical potential of species, mixture and species diffusion coefficient, respectively.

Within the DNS framework, these different entropy source terms were obtained through solving the transport equation of the specific entropy (Equation (24)). The solution of all of these aforementioned equations with DNS was performed in configurations without considering the presence of the walls [20,27]. Only recently, the effect of the wall was accounted for in [108] where the authors investigated the entropy production in the head-on interaction of turbulent premixed flames with an isothermal and chemically inert wall within turbulent boundary layers.

### 3.2. Survey of Applications Related to DNS

The DNS-based analysis can be classified in terms of the prescribed objectives. The first objective consists of providing insight into the evolution of the quantities of interest—namely, the contribution of each process to the total entropy production. Among these investigations are the ones conducted by Farran et al. [20,27] who adopted a simple chemistry for freely propagating statistically planar turbulent premixed flames. Different values of heat release parameter $\tau_{q}$, global Lewis number Le for the corrugated flamelets and thin reaction zones combustion regimes have been tested.

It was reported that the combustion regime does not significantly affect the entropy generation due to chemical reaction, thermal conduction and mass diffusion. However, for the viscous dissipation source term, the effect of combustion regime is stronger for the thin reaction zones regime compared to the corrugated flamelets one. It was found that the decrease in the global Lewis number increases the entropy generation due to heat transfer, chemical reaction and mass diffusion, unlike the heat release parameter, which only affects the heat transfer source term.

In this DNS framework, further parameter studies have been reported. These aimed at analyzing the effect of the wall boundary conditions on the different entropy generation source terms. This was achieved by Salimath et al. [109] who investigated the wall effect on the entropy production in a 1D laminar flame propagating towards and quenching at a either solid or permeable wall. Recently, Ghai et al. [108] examined the entropy production behavior in a head-on interaction of turbulent premixed methane-air flame with a chemically inert wall.

The second law analysis was performed based on three-dimensional DNS data on channel configuration in cases of isothermal and adiabatic thermal boundary conditions for the wall using a stoichiometric methane-air mixture under atmospheric conditions and a periodic boundaries conditions. As in Farran et al.’s [20,27] investigation, the combustion chemistry is represented by a single-step Arrhenius type chemical reaction. The authors revealed from this analysis that the different entropy generation source terms except the viscous term have comparable magnitudes within the flame and away from the walls, which is consistent with previous findings for turbulent premixed flames without walls [20].

However, the entropy generation due to chemical reaction decreases during flame-wall interaction (FWI) for the isothermal case due to the flame quenching as well as in the adiabatic boundary conditions due to the consumption of reactants. Moreover, in the isothermal case, the entropy generation due to thermal diffusion during the FWI is relatively higher compared to the adiabatic case in which the mean values of entropy generation due to thermal diffusion and molecular mixing have small values near the wall. Through their analysis, Ghai et al. [108] revealed the importance of the wall boundary conditions in the irreversibilities reduction and the thermodynamic performance optimization in combustion systems.

Since the entropy generation is dependent on the reaction rate and the gradients of species mass fractions and of temperature, the results that are captured here by a single-step chemistry would not be quantitatively accurate. In addition, DNS cannot be used for design and optimization tasks in turbulent combustion systems at high Reynolds numbers due to the required high computational costs even in the foreseeable future, it is advisable to rather employ DNS as a numerical experiment running at low or moderate Reynolds numbers. The gained insights can be exploited to support subgrid scale (SGS) modeling in LES for high Reynolds number flows or specific modeling in RANS.

The resulting models shall then be validated using available experimental data (possibly at high Reynolds number). The second objective of the DNS-based investigations was therefore to support the derivation of suitable RANS or/and LES models for the different entropy production contributions of the processes involved. This aspect addressed by Chakraborty [27] provided the Reynolds-averaged entropy generation models in turbulent flames. This strategy was also applied by Ries et al. [31] in LES framework but in turbulent non reacting flow, first. Some results obtained by Chakraborty [27] will be outlined in the next subsection focused on investigations using RANS models.

## 4. Numerical-Modeling-Based 3D Investigations: RANS-Based Modeling Approaches

This section provides all the classical governing equations needed to describe a reacting flow (balance equations of mass, momentum, energy and species) together with models of entropy production in the RANS context, respectively. Then, a survey of applications is delivered and discussed.

### 4.1. Governing Averaged Equations and Models

Entropy generation analysis based on the solution of the Reynolds-averaged Navier–Stokes Equations (RANS) have been conducted in many numerical studies for non-reacting flows [10,12,13,14,17,24,28,29,110]. However, because of the low predictivity in the case of complex reacting flows, limited references on entropy production evaluation based on RANS can be found [111,112,113].

Three Reynolds-averaging-based approaches were presented to model the entropy generation source terms (including in Equation (24)—namely, the Reynolds-averaging-based approach, the assumed PDF-based technique and the DNS data-based method). Differences between these approaches come out from the turbulence and combustion closure models that strongly influence the entropy generation evaluation.

#### 4.1.1. Reynolds Averaging-Based Approach

This approach uses the time-averaged of full reacting flow equations (Equations (14)–(17)), by applying the classical decomposition of the flow variable ϕ into a mean value, $\bar{\phi }$ and a fluctuating value, $\phi {}^{\prime }$. However, the Favre-averaging method is mostly preferred for turbulent reacting flows since there are large fluctuations in density, species concentrations, flow velocity and temperature:

(25) $$\phi =\tilde{\phi }+\phi {}^{\prime \prime }\;;\;\;\tilde{\phi }=\frac{\bar{\rho \phi }}{\bar{\rho }}$$

where $\phi {}^{\prime \prime }=\phi {}^{\prime }-\frac{\bar{\rho \phi }}{\bar{\rho }}$ represents the Favre fluctuation of the quantity ϕ. After averaging, the next step in modeling the flow irreversibilities is to Favre-average the instantaneous entropy Equation (Equation (24)), which leads to:

(26) $$\frac{\partial \bar{\rho }\tilde{s}}{\partial t}+\frac{\partial }{\partial x_{i}}\left[\bar{\rho }{\tilde{u}}_{i}\tilde{s}+\bar{\rho {u^{\prime \prime }}_{i}s^{\prime \prime }}-\sum_{\alpha =1}^{N_{s}}\bar{\rho }D_{\alpha }\frac{\partial {\tilde{Y}}_{\alpha }}{\partial x_{i}}{\tilde{s}}_{\alpha }-\bar{\sum_{\alpha =1}^{N_{s}}\rho D_{\alpha }\frac{\partial {Y^{\prime \prime }}_{\alpha }}{\partial x_{i}}{s^{\prime \prime }}_{\alpha }}+\bar{Q/T\;}\right]={\bar{\Pi }}_{gen}$$

The entropy generation analysis is then performed by applying the Reynolds averaging procedure to the instantaneous entropy generation rate:

(27) $${\bar{\Pi }}_{gen}={\bar{\Pi }}_{v}+{\bar{\Pi }}_{q}+{\bar{\Pi }}_{d}+{\bar{\Pi }}_{ch}$$

The averaging of each instantaneous entropy generation source term generates two components, mean and turbulent as following:

(28) $${\bar{\Pi }}_{v}≅\underset{\bar{\Pi_{v}^{M}}}{\underset{︸}{\frac{{\tilde{\tau }}_{ij}}{\tilde{T}}\frac{\partial {\tilde{u}}_{i}}{\partial x_{j}}}}+\underset{\bar{\Pi_{v}^{t}}}{\underset{︸}{\frac{\bar{\rho }\varepsilon_{K}}{\tilde{T}}}};\;\;\varepsilon_{k}=\frac{\bar{\tau_{ij}\left(\partial {u^{\prime \prime }}_{i}/\partial x_{j}\;\right)}}{\bar{\rho }}$$

(29) $${\bar{\Pi }}_{q}≅\underset{{\bar{\Pi }}_{q}^{M}}{\underset{︸}{\frac{\lambda }{{\tilde{T}}^{2}}\frac{\partial \tilde{T}}{\partial x_{i}}\frac{\partial \tilde{T}}{\partial x_{i}}}}+\underset{{\bar{\Pi }}_{q}^{t}}{\underset{︸}{\frac{\bar{\rho }c_{p}\varepsilon_{\theta }}{{\tilde{T}}^{2}}}};\;\;\varepsilon_{\theta }=\frac{\lambda }{\bar{\rho }c_{p}}\frac{\bar{\rho \left(\partial T^{\prime \prime }/\partial x_{i}\;\right)\left(\partial T^{\prime \prime }/\partial x_{i}\;\right)}}{\bar{\rho }}$$

(30) $${\bar{\Pi }}_{d}≅\underset{{\bar{\Pi }}_{q}^{M}}{\underset{︸}{\sum_{\alpha =1}^{N_{s}}\bar{\rho }D_{\alpha }\frac{R_{\alpha }}{{\tilde{Y}}_{\alpha }}\frac{\partial {\tilde{Y}}_{\alpha }}{\partial x_{i}}\frac{\partial {\tilde{Y}}_{\alpha }}{\partial x_{i}}}}+\underset{{\bar{\Pi }}_{q}^{t}}{\underset{︸}{\sum_{\alpha =1}^{N_{s}}\frac{R_{\alpha }\bar{\rho }\varepsilon_{\psi }^{\alpha }}{{\tilde{Y}}_{\alpha }}}};\;\;\varepsilon_{\psi }^{\alpha }=D_{\alpha }\bar{\left(\partial Y_{\alpha }/\partial x_{i}\;\right)\left(\partial Y_{\alpha }/\partial x_{i}\;\right)}$$

In Equations (28)–(30), $\varepsilon_{k}$ expresses the dissipation rate of the turbulent kinetic energy, $k=1/2\bar{\rho u_{i}{{}^{\prime }}^{2}}/\bar{\rho }$; $\varepsilon_{\theta }$ represents the dissipation rate of fluctuating temperature variance, $k_{\theta }=1/2\bar{\rho T{{}^{\prime \prime }}^{2}}/\bar{\rho }$, and $\varepsilon_{\psi }^{\alpha }$ is the dissipation rate of the fluctuating αth component mass fraction variance, $k_{\psi }^{\alpha }=1/2\bar{\rho Y{{}^{\prime \prime }}_{\alpha }^{2}}/\bar{\rho }$.

These formulations of the viscous, thermal and diffusion entropy generation source terms are insufficient because they require information about the mixture turbulent thermal conductivity, $\lambda_{t}$ and the turbulent mass diffusion coefficient, Dα,t of αth species. $\lambda_{t}$ and Dα,t can be derived from the turbulent Prandtl number, Pr_t_ and the turbulent Schmidt number, Sc_t_. Thus, the determination of the turbulent thermal and diffusion entropy generation sources terms is achieved by either assuming constant Pr_t_ and Sc_t_ or non constant ones computed with some algebraic relations [111]:

(31) $${\bar{\Pi }}_{q}^{t}=\frac{\bar{\rho }c_{p}\varepsilon_{\theta }}{{\tilde{T}}^{2}}≅\frac{\lambda_{t}}{{\tilde{T}}^{2}}\frac{\partial \tilde{T}}{\partial x_{i}}\frac{\partial \tilde{T}}{\partial x_{i}}$$

(32) $${\bar{\Pi }}_{d}^{t}=\sum_{k=1}^{N_{s}}\frac{R_{k}\bar{\rho }\varepsilon_{\psi }^{k}}{{\tilde{Y}}_{k}}≅\bar{\rho }D_{t}\sum_{k=1}^{N_{s}}\frac{R_{k}}{{\tilde{Y}}_{k}}\frac{\partial {\tilde{Y}}_{k}}{\partial x_{i}}\frac{\partial {\tilde{Y}}_{k}}{\partial x_{i}}$$

If transport equations of $k_{\theta }$ and $k_{\psi }^{\alpha }$ are accounted for in the RANS modeling (one-equation model concept), the previous approximations (Equations (31) and (32), will be useless since the dissipation rate of fluctuating temperature and mass fraction of αth species variances $\varepsilon_{\theta }$, $\varepsilon_{\psi }^{\alpha }$, respectively, will be available. For the remaining entropy generation source term that represents the contribution of the chemical reaction, only the mean entropy generation source term is available. It is computed by applying the average of the full reaction rate ω of the reacting flow as follow [111]:

(33) $${\bar{\Pi }}_{ch}≅\frac{\tilde{\omega }}{\tilde{T}}\sum_{\alpha =1}^{N_{s}}\left({\upsilon^{\prime }}_{\alpha }-{\upsilon^{\prime \prime }}_{\alpha }\right){\tilde{\mu }}_{M,\alpha };\;\;{\tilde{\mu }}_{M,\alpha }={\tilde{h}}_{M,\alpha }-\tilde{T}s_{M,\alpha }\left(\tilde{T},\tilde{p},{\tilde{X}}_{\alpha }\right)$$

where $\mu_{M,\alpha }$, $h_{M,\alpha }$ and $s_{M,\alpha }$ are the mean chemical potential, specific enthalpy and specific entropy of the $\alpha^{th}$ species, respectively.

#### 4.1.2. Assumed Probability Density-Based Approach

In contrast with the previous approach, the turbulence–chemistry interaction will be explicitly accounted for. Within the RANS modeling framework, this is achieved by coupling the RANS model equations with the probability density function technique (PDF). The objective is here to provide the probability distribution of a certain property of the reacting flow at a given location in the flow system. In the case of non-premixed reacting flow system the probability distribution is given by the mean and variance of the mixture fraction at each point of the reacting flow. Any needed mean thermo-chemical properties are then computed according to:

(34) $$\tilde{\phi }=\int_{0}^{1}\phi \left(Z\right)\tilde{f}\left(Z\right)dZ$$

where ϕ(*Z*) is a generic instantaneous thermo-chemical flow variable derived from the mixture fraction solution using the Burke-Schumann approximation [114] and f(Z) is the PDF of the mixture fraction. The PDF methods are either assumed (presumed) or transported. Stanciu et al. [111] considered a presumed PDF (a Gaussian distribution) for diffusion flame and computed the entropy generation source terms as:

(35) $${\bar{\Pi }}_{q}=\frac{\bar{\rho }}{2}\tilde{\chi }\int_{0}^{1}\rho c_{p}{\left(\frac{dT}{dZ}\right)}^{2}\frac{f\left(Z\right)}{T^{2}\left(Z\right)}dZ$$

(36) $${\bar{\Pi }}_{d}=\frac{\bar{\rho }}{2}\tilde{\chi }\int_{0}^{1}\left[\sum_{\alpha =1}^{N_{s}}\frac{R_{\alpha }}{Y_{\alpha }}{\left(\frac{dY_{\alpha }}{dZ}\right)}^{2}\right]f\left(Z\right)dZ$$

(37) $${\bar{\Pi }}_{ch}=\frac{\bar{\rho }}{2}\tilde{\chi }\int_{0}^{1}\left[\frac{1}{T\left(Z\right)}\sum_{\alpha =1}^{N_{s}}\mu_{\alpha }\left(Z\right)\frac{d^{2}Y_{\alpha }}{dZ^{2}}\right]f\left(Z\right)dZ$$

Equations (35)–(37) are derived under consideration of the unity Lewis number, where $D_{\alpha }$ (α = 1, …, $N_{s}$) are mixture diffusion coefficients, $D_{\alpha }=D$. The quantity $\tilde{\chi }$ represents the Favre-averaged scalar dissipation rate given as:

(38) $$\tilde{\chi }=\frac{2}{\bar{\rho }}\left[\bar{\rho D\frac{\partial Z^{\prime \prime }}{\partial x_{i}}\frac{\partial Z^{\prime \prime }}{\partial x_{i}}}\right]=2D\frac{\partial \tilde{Z}}{\partial x_{i}}\frac{\partial \tilde{Z}}{\partial x_{i}}$$

Finally, the viscous entropy generation source term was calculated in the same way as the previous approach (Equation (28)).

#### 4.1.3. DNS Data-Based Approach

In this approach, the Reynolds-averaged entropy generation source terms are obtained from an a priori analysis of DNS data. New models to predict the different entropy production source terms are derived from direct numerical simulation (DNS) database within a range of different operating parameters. Chakraborty [27] attempted to model these source terms for a freely propagating statistically planar turbulent premixed flame in both corrugated flamelets (CF) and thin reaction zones (TRZ) regimes within the RANS context. They utilized data from DNS performed for different values of heat release parameter $\tau_{q}$, global Lewis number Le and turbulent Reynolds number Re_t_. Note that for sake of simplicity, a single-step Arrhenius type chemistry was used. Starting with the viscous dissipation contribution, the entropy generation term is modeled as:

(39) $${\bar{\Pi }}_{v}=\frac{\tau_{ij}^{R}}{\tilde{T}}\left(\frac{\partial {\tilde{u}}_{i}}{\partial x_{j}}\right)+C_{\Pi_{v}}c_{p}\left(\gamma -1\right)Ma_{L}^{2}\frac{\bar{\rho }{\tilde{\varepsilon }}_{k}}{S_{L}^{2}}$$

where $\tau_{ij}^{R}$ is the resolved viscous stress tensor, γ is the ratio of specific heats ($\gamma =c_{p}/c_{v}$), $S_{L}$ is the unstrained laminar burning velocity and $Ma_{L}^{2}$ is the local Mach number ($Ma_{L}=S_{L}/\sqrt{\gamma RT_{0}\left(1+\tau_{q}{\tilde{Y}}_{c}\right)}$). Accurate prediction of the viscous entropy generation source term in RANS context compared to DNS was found when setting the model parameter, $C_{\Pi_{v}}$ equal to 1.

The Reynolds-averaged heat transfer entropy generation source term was modeled as:

(40) $${\bar{\Pi }}_{q}=\frac{\lambda \nabla \tilde{T}\cdot \nabla \tilde{T}}{{\tilde{T}}^{2}}+C_{\Pi_{q}}c_{p}\bar{\rho }{\tilde{\varepsilon }}_{Y_{c}}\;;\;C_{\Pi_{q}}=4.4{\left[\tau_{q}/\left(1-\tau_{q}\right)\;\right]}^{3.63}$$

where $\varepsilon_{c}=\bar{\rho D\nabla Y_{c}\cdot \nabla Y_{c}}/\bar{\rho }\;-\tilde{D}\nabla {\tilde{Y}}_{c}\cdot \nabla {\tilde{Y}}_{c}$ and the used model parameter $C_{\Pi_{q}}$ expressed in Equation (40) leads to a satisfactory quantitative prediction of ${\bar{\Pi }}_{q}$.

The derived model of Reynolds-averaged mass diffusion entropy source term from DNS data [27] is expressed as:

(41) $${\bar{\Pi }}_{d}=\left(1-\varphi^{3}\right)\sum_{\alpha =1}^{N_{s}}\frac{\bar{\rho }\tilde{D}R_{\alpha }\nabla {\tilde{X}}_{\alpha }\cdot \nabla {\tilde{Y}}_{\alpha }}{{\tilde{X}}_{\alpha }}+C_{\Pi_{d}}c_{p}\bar{\rho }\sum_{\alpha =1}^{N_{s}}{\tilde{\varepsilon }}_{\alpha }\;;\;\varphi =\frac{{\tilde{\varepsilon }}_{Y_{c}}}{\left({\tilde{\varepsilon }}_{c}+\tilde{D}\nabla {\tilde{Y}}_{c}\cdot \nabla {\tilde{Y}}_{c}\right)},$$

where the quantity φ expresses a measure of unresolvedness while $C_{\Pi_{d}}$ represents a model parameter. For $C_{\Pi_{d}}=1$, this model accurately predict the mass diffusion entropy source term.

Lastly, the chemical reaction entropy generation source term model within the RANS context is given as:

(42) $${\bar{\Pi }}_{ch}=f_{1}\left(\varphi \right)\left[-\sum_{\alpha =1}^{N_{s}}\frac{\mu_{\alpha }^{R}{\bar{\dot{\omega }}}_{\alpha }}{\tilde{T}}\right]+f_{2}\left(\varphi \right)C_{\Pi_{ch}}c_{p}\bar{\dot{\omega }}$$

In Equation (42), $\mu_{\alpha }^{R}$ is the resolved chemical potential defined as a function of Favre mean quantities (i.e., $\mu_{\alpha }^{R}=f\left({\tilde{h}}_{\alpha },{\tilde{Y}}_{1},\ldots ,{\tilde{Y}}_{Ns}\right)$). $\dot{\omega }$ is the mean reaction progress (defined here as $\dot{\omega }=\rho_{0}S_{L}^{2}/\alpha_{T}$, where $\rho_{0}$ and $\alpha_{T}$ are the density and thermal diffusivity of the unburned gas, respectively). Better results were obtained by choosing the model parameters $C_{\Pi_{ch}}$ and $f_{1}$, $f_{2}$ as follows:

(43) $$f_{1}\left(\varphi \right)=\left(1-\varphi^{3}\right);\;f_{2}\left(\varphi \right)=\varphi^{1/4\;};\;C_{\Pi_{ch}}=\;0.4Le^{0.64}\;$$

### 4.2. Survey of Applications Using the RANS Approach

As detailed in Section 4, three different approaches for entropy generation analysis in the RANS context can be found in the literature. The Reynolds-averaging-based approach and the assumed probability-density-based approach were both intensively investigated by Stanciu et al. [111]. They applied these approaches on a Delft piloted diffusion flame burner [115] that was designed to yield a stable axial-symmetric turbulent non-premixed flame of methane burning in a co-flowing air stream.

Table 1 presents the integrated values of entropy generation source terms over the combustion chamber volume and the related closing error once the RNG k-epsilon model is used. The heat transfer and the chemical reaction are found as the main responsible of entropy generation with a contribution of 38% and 57%, respectively. Compared to the statement reported in the literature [32,33], the chemical source term is over-predicted due to the consideration of the eddy break-up model [111,116], which is known for its overestimation of the the mean reaction rate.

Modeling the entropy generation based on the assumed PDF coupled with RANS turbulence models was also investigated by Stanciu et al. [111] as well as by Nadim [115]. The latter applied the entropy analysis on Sandia H2-A flame, which is a diffusion flame consisting of a jet with 100% of H2 in composition. For the RANS simulations, the following assumptions were considered: (1) The axial variation in mixture fraction, *Z* is assumed to be the same for the whole radial cross section of the flame. (2) Independent scalar dissipation rate, χ, of the other variables is considered. (3) The mean entropy generation is computed in 2D. (4) A single step reaction mechanism of H2 combustion is used. (5) Although H2 flame is under study, the differential diffusion is not accounted for by assuming the Lewis number to be unity.

The author revealed that the heat transfer and chemical reaction featured the major contributions on the entropy production. It was also reported that the viscous dissipation source term is neglected and the percentages of contribution in entropy generation are 38.5%, 37.4% and 24.1% for the heat transfer, chemical reaction and mass diffusion, respectively. The same approach was also adopted by Tehrani et al. [113] to determine the relation between the soot formation and the entropy production in a turbulent Kerosene/Air jet diffusion flames using the β-PDF distribution, the laminar flamelet model for combustion and the Moss–Brookes–Hall (MBH) [117] model for soot formation.

All simulations were performed in a combustion chamber made of borosilicate glass-tube under adiabatic conditions but with consideration of the heat radiation. From this analysis it is reported that the maximum generated entropy is reached when the soot formation is completed and that the entropy generation as well as the temperature are inversely related to the soot formation. It was found, that the entropy production is more related to the temperature than to the soot formation rate. Through their investigation, Tehrani et al. [113] showed that the entropy production can predict the main region of soot formation with a tolerable deviation.

Despite the simplicity and the introduced assumed form of the pdf, it remains difficult to judge the accuracy of the obtained results with this approach. In addition to the dissipative character of RANS, many assumptions were considered during the simulations, among them the unity Lewis number for H2 flame and the simple chemistry mechanism in [115], which can significantly affect the results.

To face the encountered modeling challenges while quantifying the different entropy production source terms within the RANS context, Chakraborty [27] exploited their DNS data to derive models for the Reynolds-averaged entropy generation in turbulent flames. This strategy was also applied by Ries et al. [31] to suggest LES models first in turbulent non reacting flows. Focused on the effort by Chakraborty [27], the entropy generation source terms were modeled using Equations (26)–(29) derived from DNS data for different operating conditions (varied Re, *Le* and $\tau_{q}$).

The obtained models were validated against DNS results, and the suitable model constants, $C_{\Pi_{v}}$, $C_{\Pi_{q}}$, $C_{\Pi_{d}}$ and $C_{\Pi_{ch}}$ were consistently determined. The proposed models effectively reproduce the entropy generation achieved by DNS even though the DNS data (which are generated using a simple reaction mechanism) cannot quantitatively describe the entropy generation in an accurate way as previously detailed in Section 3.1.

In addition to the efforts made to investigate generic or canonical systems, numerical investigations on entropy generation in the RANS framework were also performed in practical systems, such as IC engines [118,119,120,121,122]. Most of the reported numerical studies related to IC engines extend to 3D the methodology referenced above in the experimental investigations [37,39,41,43,44,46] mentioned in the previous section dealing with simple exergy or energy balance.

Jafamrmadar [118] applied exergy analysis in pre- and main chambers of a Lister 8.1 indirect injection diesel engine (IDI) for two loads (50% and full-load) operating conditions. Numerical simulations were performed using CFD code using RNG k–ϵ turbulence model and the Reynolds-averaged data obtained from flow simulation were used to calculate the different exergy terms, including fuel, heat loss, irreversibility, work, exhaust loss, chemical and thermo-mechanical exergy contributions in both the pre- and main chambers of the engine.

The author reported that the main combustion chamber features 77.4% and 55.7% of total irreversibility at part and full load operations, respectively. Through this work, they found that multidimensional modeling can be useful in complex chamber configurations: based on the second law of thermodynamics, this can enables greater insight into the effect of the flow fields on the combustion process. In their next paper, Jafamrmadar [119] applied second law analysis on a Deutz dual fuel (diesel+hydrogen) engine at different gas fuel–air ratios. He found that by increasing the gas fuel–air equivalence ratio from 0.3 to 0.8, the exergy efficiency decreases from 43.7% to 34.5%.

Recently, Fathi et al. [121] and Mondo et al. [122] also applied the exergy balance within the RANS framework on an IC engine. The first investigation [121] revealed that the variation of the spray angle in a Reactivity Controlled Combustion Ignition (RCCI) dual fuel engine, fueled by n-heptane, affects the exergy efficiency, which found its maximum value for spray angle between 5° and 20°. The second work [122] performed the investigation on direct-injection (DI), single-cylinder and four-stroke diesel engines to test different fuel reaction mechanisms, which are numerically generated and assess their predictability in comparison with available experimental data. The exergy efficiency was found to be different when using different reaction mechanisms and was observed to follow the behaviors of the pressure variations.

Considerations of thermal radiation received particular attention due to direct relevance of radiation in a variety of engineering and technical systems operating at high temperatures. Industrial furnaces, heat exchangers, heat storage, solar collectors, transport in packed and circulating bed combustors, and reactors, manufacturing and materials processing, oil reservoirs and thermal insulation applications can be mentioned among others. However, only few contributions have been devoted to include numerically unsteady multidimensional thermal problems, including thermal radiation, into Entropy Generation Analysis [123,124,125].

To sum up, the application of the entropy generation analysis in the RANS context does not seem promising, since RANS model is dissipative by nature and cannot provide accurate information concerning the unsteady behavior of different processes involved in combustion at the subgrid level, where the major system irreversibilities occur. In additions, the attempts found in the literature [27,111,115] to ameliorate second law analysis in the RANS context are not enough as they implied several simplification assumptions, which significantly affect the results.

Finally, numerical investigations based on second law analysis in the RANS context, summarized also in Table A1 (see Appendix A), are mostly restricted to simple exergy balance in order to compute the exergy efficiency, sometimes without calculating the different exergy losses, their distributions throughout the system and their relative contributions in the total exergy loss.

## 5. Numerical-Modeling-Based 3D Investigations: LES-Based Modeling Approaches

This section first provides all the governing filtered equations in the frame of the classical LES needed to describe a reacting flow (balance equations of mass, momentum, energy and species) together with subgrid scale models of entropy production in the LES context. A survey of applications is thereafter presented and discussed.

Since direct numerical simulation (DNS) is still expensive in terms of computational cost despite the evolution of the high-computing performance and the RANS-based modeling is not accurate enough for turbulent reacting flows, large eddy simulation (LES) seems to be suitable to accurately perform the entropy generation analysis as testified by [15,23,31] who reported appreciable comparisons with DNS data. The entropy production is predominantly a subgrid-scale process since it is related to the dissipation of energy, and therefore accurate closure approaches were required. In turbulent reacting flows, the turbulence-chemistry interaction, which occurs at the sub-grid scale must additionally be considered to correctly represent the thermo-chemical quantities, which in turn allows to reduce uncertainty in the evaluation of the entropy production terms of the various processes.

Nevertheless, considering the detailed chemistry within the LES framework through a chemistry tabulation reduction technique makes it possible to rather use LES instead of DNS even for complex reacting flow systems. In the literature, the Flamelet-Generated Manifold (FGM) was adopted as one of the most promising reduction strategies, since it enables to describe the detailed chemistry with only a few control parameters, generally the mixture fraction and a reaction progress variable for adiabatic flow systems.

### 5.1. Governing Filtered Equations and Subgrid Scale Models

First, this subsection provides all the classical governing equations needed to describe an adiabatic reacting flow and entropy generation according to FGM-based combustion model within the LES framework. The filtered balance equations to be solved are:

(44) $$\frac{\partial \bar{\rho }}{\partial t}+\frac{\partial \bar{\rho }{\tilde{u}}_{i}}{\partial x_{i}}=0$$

(45) $$\frac{\partial \bar{\rho }{\tilde{u}}_{i}}{\partial t}+\frac{\partial \bar{\rho }{\tilde{u}}_{i}{\tilde{u}}_{j}}{\partial x_{j}}=-\frac{\partial \bar{p}}{\partial x_{i}}+\frac{\partial }{\partial x_{j}}\left[\bar{\mu }\left(\frac{\partial {\tilde{u}}_{i}}{\partial x_{j}}+\frac{\partial {\tilde{u}}_{j}}{\partial x_{i}}-\frac{2}{3}\frac{\partial {\tilde{u}}_{k}}{\partial x_{k}}\delta_{ij}\right)\right]\_\frac{\partial }{\partial x_{j}}\left(\bar{\rho }\tau_{ij}^{sgs}\right)$$

(46) $$\frac{\partial \bar{\rho }\tilde{Z}}{\partial t}+\frac{\partial \bar{\rho }{\tilde{u}}_{j}\tilde{Z}}{\partial x_{j}}=\frac{\partial }{\partial x_{j}}\left[\left(\frac{\bar{\mu }}{Sc}+\frac{\mu_{sgs}}{Sc_{sgs}}\right)\frac{\partial \tilde{Z}}{\partial x_{j}}\right]$$

(47) $$\frac{\partial \bar{\rho }{\tilde{Y}}_{c}}{\partial t}+\frac{\partial \bar{\rho }{\tilde{u}}_{j}{\tilde{Y}}_{c}}{\partial x_{j}}=\frac{\partial }{\partial x_{j}}\left[\left(\frac{\bar{\mu }}{Sc}+\frac{\mu_{sgs}}{Sc_{sgs}}\right)\frac{\partial {\tilde{Y}}_{c}}{\partial x_{j}}\right]+{\bar{\dot{\omega }}}_{Yc}$$

(48) $$\begin{array}{cc} \; & \frac{\partial \bar{\rho }\tilde{s}}{\partial t}+\frac{\partial }{\partial x_{i}}\left(\bar{\rho }{\tilde{u}}_{i}\tilde{s}\right)=\frac{\partial }{\partial x_{i}}\left(\bar{\rho }D_{m}\frac{\partial \tilde{s}}{\partial x_{i}}\right)-\frac{\partial }{\partial x_{i}}\left(\bar{\rho }\tau \left(u_{i},s\right)\right)\\ \; & +\underset{\Pi_{v}}{\underset{︸}{\bar{\frac{1}{T}\tau_{ij}\frac{\partial u_{i}}{\partial x_{j}}}}}+\underset{\Pi_{q}}{\underset{︸}{\bar{\frac{\lambda }{T^{2}}\frac{\partial T}{\partial x_{i}}\frac{\partial T}{\partial x_{i}}}}}+\underset{\Pi_{d}}{\underset{︸}{\bar{\frac{\lambda }{c_{p}}\sum_{\alpha =1}^{N}\frac{R_{\alpha }}{Y_{\alpha }}\frac{\partial Y_{\alpha }}{\partial x_{i}}\frac{\partial Y_{\alpha }}{\partial x_{i}}}}}-\underset{\Pi_{ch}}{\underset{︸}{\bar{\frac{1}{T}\sum_{\alpha =1}^{N}\mu_{\alpha }{\dot{\omega }}_{\alpha }}}} \end{array}$$

In Equations (44)–(48), the notations $\bar{\left(.\right)}$, $\tilde{\left(.\right)}$ and (.)*_sgs_* stand for the filtered, Favre-filtered and sub-grid scale quantities, respectively. The filtered transport equations for the control variables (Equations (46) and (47)) are solved together with the classical filtered transport equations for mass density (Equation (44)) and momentum (Equation (45)). Note that the quantity $\delta_{ij}$ is the Kronecker delta and $\tau_{ij}$ the sub-grid scale stress tensor.

The latter may be closed by means of a chosen eddy-viscosity turbulence model. The reaction source term, $\dot{\omega }Yc$, in Equation (47) remains normally unclosed in the LES context. Recently, an approach that relies on a transport equation of the Filtered Density Function (FDF) as adopted in [126,127], is known to provide the chemical source term in a closed form.

The entropy generation evaluation is thereby conducted either by solving the whole entropy transport Equation (Equation (48)) together with the set of Equations (44)–(47) or only by modeling the four entropy generation source terms, including in Equation (48). Consequently, the total entropy generation, $S_{gen}$, which is the sum of these source terms can be obtained and the irreversibility ratio of each process involved in entropy production can be evaluated as:

(49) $$\Psi_{v}=\frac{\Pi_{v}}{S_{gen}};\;\;\;\;\;\Psi_{q}=\frac{\Pi_{q}}{S_{gen}};\;\;\;\;\Psi_{d}=\frac{\Pi_{d}}{S_{gen}};\;\;\;\;\;\Psi_{ch}=\frac{\Pi_{ch}}{S_{gen}}$$

In the literature, three approaches for the entropy generation source terms evaluation have been suggested [31,32,33,128,129]. They are summarized in the next subsections.

#### 5.1.1. Thermodynamics-Based Approach

The first studies performed on entropy generation analysis using LES were reported by Safari et al. [32,128,129]. They proposed and used an approach that relies on a transport equation of the Filtered Density Function (FDF) of entropy in addition to the classical filtered governing equations. To formulate models for the non-closed terms, the authors applied the methodology based on the Transported Filtered Density Function (T-FDF), which provides the chemical reaction term and its entropy generation contribution in closed forms (see ($\Pi_{ch}$) in Equation (48)).

In particular, the FDF transport equation is modeled by a set of Stochastic Differential Equations (SDEs) corresponding to position, scalars and entropy [32]. Using these SDEs together with the classical Gibbs relation [130], and following the indications detailed in [131,132,133], the models of the non-closed terms of entropy generation in Equation (48) emerge as:

(50) $${\bar{\Pi }}_{v}\approx \bar{\rho }\frac{1}{\tilde{T}}\varepsilon_{t};{\bar{\Pi }}_{q}\approx \frac{\bar{\rho }}{\tau_{t}}\left[\sum_{\alpha =1}^{Ns}\tau \left(Y_{\alpha },\frac{g_{\alpha }}{T}\right)-\tau \left(h,\frac{1}{T}\right)\right];{\bar{\Pi }}_{d}\approx \frac{\bar{\rho }}{\tau_{t}}\sum_{\alpha =1}^{Ns}R_{\alpha }\tau \left(Y_{\alpha },lnX_{\alpha }\right)$$

where, in the entropy production term due to viscous dissipation $\Pi_{v}$ (Equation (50)), $\varepsilon_{t}$ stands for the total rate of turbulent dissipation. It includes both sub-grid scale (by means of SGS kinetic energy, *k_sgs_*, and the sub-grid mixing time scale, $\tau_{t}$) and resolved contributions. It is given by [32,128,129]:

(51) $$\varepsilon_{t}=\frac{k_{sgs}}{C_{\Omega }\tau_{t}}+\frac{1}{\bar{\rho }}{\bar{\tau }}_{ij}\frac{\partial {\tilde{u}}_{i}}{\partial x_{j}}$$

In Equation (50), $g_{\alpha }$ and $X_{\alpha }$ are the specific Gibbs free energy and the mole fraction of the species α, respectively.

#### 5.1.2. Turbulence-Based Approach

According to the investigation in the non-reacting flow by Ries et al. [31], the entropy generation analysis is performed without the solution of the transport equation of entropy. The entropy production source terms were rather computed in a post-processing phase of LES. Thereby, the effects of sgs entropy generation are modeled by simple algebraic equations based on resolved turbulent quantities. As indicated above, the proposed approach is computationally inexpensive and can be used as a simple post-processing tool. In addition, this approach can be easily applied with existing eddy-viscosity-based models. Following the classical decomposition in resolved and SGS contributions for each term as:

(52) $$\Pi_{j}=\Pi_{j}^{res}+\Pi_{j}^{sgs}\;\mathrm{with}\;j=\left\{v,q,d,ch\right\}$$

The unclosed entropy production terms can then be approximated as:

(53) $${\bar{\Pi }}_{v}=\underset{\Pi_{v}^{res}}{\underset{︸}{\frac{\bar{\rho }\bar{\nu }}{\bar{T}}\left(\frac{\partial {\bar{u}}_{i}}{\partial x_{j}}+\frac{\partial {\bar{u}}_{j}}{\partial x_{i}}\right)\frac{\partial {\bar{u}}_{i}}{\partial x_{j}}}}+\underset{\Pi_{v}^{sgs}}{\underset{︸}{\frac{\bar{\rho }}{\bar{T}}\epsilon_{k,sgs}\;}}\;;{\bar{\Pi }}_{q}=\underset{\Pi_{q}^{res}}{\underset{︸}{\frac{\bar{\lambda }}{{\bar{T}}^{2}}\frac{\partial \bar{T}}{\partial x_{i}}\frac{\partial \bar{T}}{\partial x_{i}}}}+\underset{\Pi_{q}^{sgs}}{\underset{︸}{\frac{\bar{\rho }{\bar{c}}_{p}}{{\bar{T}}^{2}}\epsilon_{\theta ,sgs}}}$$

(54) $${\bar{\Pi }}_{d}=\underset{\Pi_{d}^{res}}{\underset{︸}{\frac{\bar{\mu }}{\bar{S}c}\sum_{\alpha =1}^{Ns}\frac{R_{\alpha }}{{\bar{Y}}_{\alpha }}\frac{\partial {\bar{Y}}_{\alpha }}{\partial x_{i}}\frac{\partial {\bar{Y}}_{\alpha }}{\partial x_{i}}}}+\underset{\Pi_{d}^{sgs}}{\underset{︸}{\sum_{\alpha =1}^{Ns}\frac{R_{\alpha }\bar{\rho }}{{\bar{Y}}_{\alpha }}\frac{2}{3C_{OC}\pi^{4/3}C_{s}^{4/3}}\frac{\upsilon_{sgs}}{Sc}\frac{\partial {\bar{Y}}_{\alpha }}{\partial x_{i}}\frac{\partial {\bar{Y}}_{\alpha }}{\partial x_{i}}}}$$

In Equation (53), $\epsilon_{k,sgs}$ and $\epsilon_{\theta ,sgs}$ stand for the dissipation rate of the SGS turbulent kinetic energy and temperature variance. Following [31,134,135], the dissipation rate of the SGS kinetic energy is given as:

(55) $$\epsilon_{k,sgs}=\frac{1}{\Delta^{4}C_{s}^{4}}{\upsilon_{sgs}}^{3}$$

where *C_s_* = 0.17, is the Smagorinsky constant. In particular, $\epsilon_{\theta ,sgs}$ is obtained from scaling the Obukhov–Corrsin inertial-convective subrange [31,136] as:

(56) $$\epsilon_{\theta ,sgs}=\frac{4}{3C_{OC}\pi^{4/3}C_{s}^{4/3}}\frac{\upsilon_{sgs}}{\mathrm{Pr}}\frac{\partial \bar{T}}{\partial x_{i}}\frac{\partial \bar{T}}{\partial x_{i}}$$

where *C_OC_* = 1.34 is the coefficient of the 3D temperature spectrum [135]. Note that Equation (54) includes a similar term for the species mass fraction variance (the last term). Adoptng the T-FDF method to account for the turbulence–chemistry interaction in coupling with the FGM-based tabulated detailed chemistry, the chemical reaction contribution to entropy production, Π*_ch_*, is expressed in the same way as in the first approach (last term on the RHS of Equation (48).

Thus, the chemical source term and the associated chemical reaction contribution to the entropy generation appear in an exact form. By the way, the solution of the T-FDF equations of the controlling variables provides the filtered mean and sub-grid variance of Π*_ch_* through the first and second moments.

#### 5.1.3. Look-Up-Table-Based Approach

This strategy introduced by Agrebi et al. [33] focuses on an efficient use of the tabulation chemistry technique. Thereby, the different entropy generation source terms are calculated in the post-process step while preparing the FGM manifold. These quantities are then stored in the look-up table, as are thermo-chemical properties needed for the combustion simulation. Since all the thermo-chemical quantities are solely function of the controlling variables, one applies the partial differentiation rule to calculate the derivatives appearing in different entropy production source terms.

In the case of an adiabatic combustion system, a 2D-FGM manifold is spanned by means of the two controlling variables—namely, the mixture fraction *Z* and the progress variable *Y_c_*—and thus the entropy production source terms are expressed as follows:

(57) $$\Pi_{q}=\frac{\lambda }{T^{2}}\frac{\partial T}{\partial x_{i}}\frac{\partial T}{\partial x_{i}}=\frac{\lambda }{T^{2}}\left[{\left(\frac{\partial T}{\partial Y_{c}}\frac{\partial Y_{c}}{\partial x_{i}}\right)}^{2}+2\frac{\partial T}{\partial Y_{c}}\frac{\partial T}{\partial Z}\frac{\partial Y_{c}}{\partial x_{i}}\frac{\partial Z}{\partial x_{i}}+{\left(\frac{\partial T}{\partial Z}\frac{\partial Z}{\partial x_{i}}\right)}^{2}\right]$$

(58) $$\Pi_{d}=\frac{\lambda }{c_{p}}\sum_{k=1}^{N}\frac{R_{k}}{Y_{k}}\frac{\partial Y_{k}}{\partial x_{i}}\frac{\partial Y_{k}}{\partial x_{i}}=\frac{\lambda }{c_{p}}\left[\begin{array}{cc} \; & \sum_{k=1}^{N}\frac{R_{k}}{Y_{k}}{\left(\frac{\partial Y_{k}}{\partial Y_{c}}\frac{\partial Y_{c}}{\partial x_{i}}\right)}^{2}+\sum_{k=1}^{N}2\frac{R_{k}}{Y_{k}}\frac{\partial Y_{k}}{\partial Y_{c}}\frac{\partial Y_{k}}{\partial Z}\frac{\partial Y_{c}}{\partial x_{i}}\frac{\partial Z}{\partial x_{i}}\\ \; & +\sum_{k=1}^{N}\frac{R_{k}}{Y_{k}}{\left(\frac{\partial Y_{k}}{\partial Z}\frac{\partial Z}{\partial x_{i}}\right)}^{2} \end{array}\right]$$

(59) $$\Pi_{ch}=-\frac{1}{T}\sum_{k=1}^{N}\mu_{k}{\dot{\omega }}_{k}$$

Note that the entropy production from viscous dissipation for this approach is calculated in the same way as in the first and second approaches (see Equations (50) and (53)).

### 5.2. Survey of Applications Relying on LES

Investigations on Entropy generation analysis based on the classical LES framework that accounts for the subgrid-scale entropy production rates are scarcely available in the literature, Safari et al. [32] were the first to report on an EGA contribution dealing with LES. They relied on a transport equation of the Filtered Density Function (FDF) of entropy together with the classical filtered governing equations that facilitate LES modeling of entropy transport and production in turbulent reacting flows. In particular, the FDF transport equation was modeled by a set of Stochastic Differential Equations (SDEs) corresponding to the position, scalars and entropy [32].

They applied this methodology to estimate entropy production in a turbulent mixing layer and a turbulent non-premixed piloted methane jet flame (so-called Sandia flame D [137]). Safari et al. [32] reported that heat transfer and chemical reaction are the most dominant modes of entropy production in this flame D. Notwithstanding this performance, the main drawback of this approach is its expensive computational cost. This method was denoted later by Agrebi et al. [33] as the thermodynamics-based approach.

To address the issue experienced by the approach by Safari et al. [32], two novel techniques were proposed by Agrebi et al. [33] who based their work on the analysis in [32] while following the Ries et al. [31] methodology demonstrated in turbulent non reacting flow to quantify the entropy generation sources. The new approaches were referred to as turbulence-based and Look-up table approaches (see Section 5.1). Agrebi et al. [33] evaluated the three different approaches (thermodynamics-based approach, turbulence-based method and look-up table ansatz) in the Sandia flame D and E while applying the hybrid filtered Eulerian stochastic field (ESF) method coupled with the flamelet generated manifold (FGM) in the LES framework.

The Sandia flames D and E are diffusion methane flames (25% CH4 and 75% air) with the same jet composition but different inlet velocities leading to higher Re in flame E than in flame D, see in Agrebi et al. [33]. The numerical simulations were performed using structured mesh refined near the piloted CH4/Air jet flames configuration’s walls and in the main jet where strong gradients of velocity, temperature, mass species, etc. are present.

**Figure 4:**
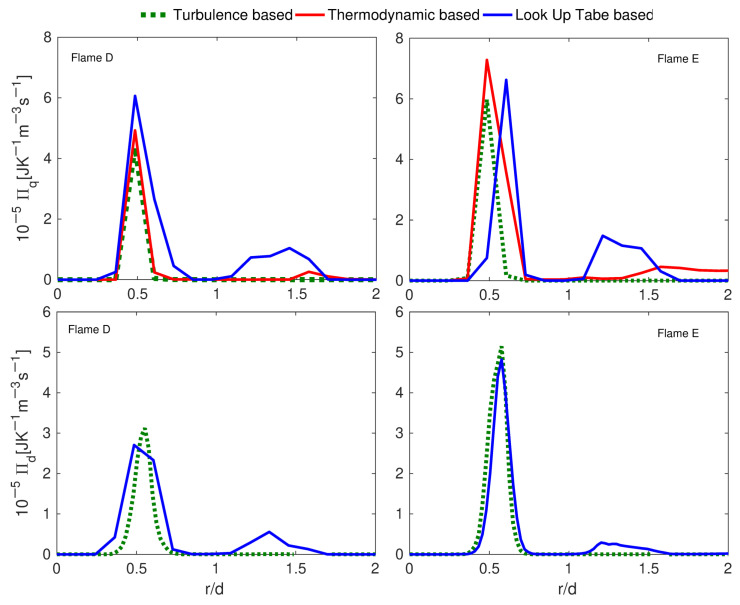
Radial profile of the volumetric entropy from heat transfer (**top**) and mass diffusion (**bottom**): Comparison of three approaches (thermodynamics-based, turbulence-based and look-up-table-based approach) at the 1D axial position for flame D (**left**) and flame E (**right**) (modified from [33,138]).

The first step was to compare and validate the achievements with respect to the entropy generation source terms using the outcome from Safari et al. [32] for Sandia flame D. Figure 4 shows that the three approaches deliver similar results and comparable to those from Safari et al. [32]. In particular, the new approaches reduce the computational cost for the entropy analysis especially the look-up-table-based approach to the order of 20-times less than the thermodynamics-based method.

**Figure 5:**
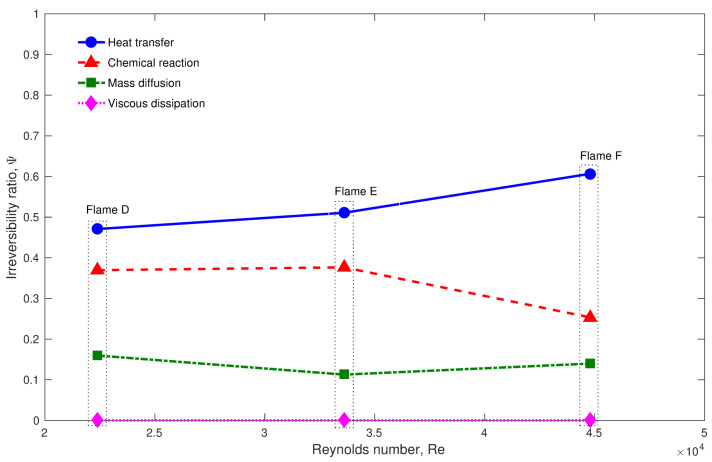
Irreversibility ratio of heat transfer, chemical reaction, mass diffusion and viscous dissipation processes as a function of the Re-number for Sandia flames D, E and F obtained by using the look-up-table-based method (modified from [33,138]).

**Figure 6:**
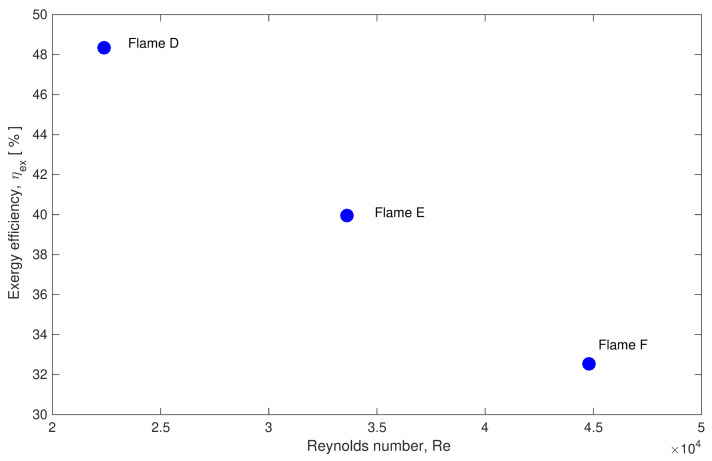
Comparison of exergy efficiency of Sandia flames as function of Re-number obtained using the look-up-table-based method (modified from [33,138]).

The second step was to analyze the features of the entropy production in Sandia flames in terms of quantitative prediction values. Thus, the application of the look-Up table based approach was recently extended by Agrebi et al. [138] to flame F. It was found that the entropy generation increases with the Reynolds number (From flame D to F). Similar to Safari et al.’s [32] work, the heat transfer and the chemical emerge as the main responsible of the entropy generation for all flames as depicted in Figure 5, which displays the irreversibility ratios for each involved process for Sandia flames D, E and F. Since the entropy generation increases with Reynolds number (From flame D to F), it is found that the exergy efficiency decreases as shown in Figure 6.

Agrebi et al. [138], while investigating flame E and F, included the exhaust gas exergy and figure out the following three conclusions: (1) the chemical exergy content of exhaust gases decreases going towards the combustion chamber outlet, (2) the temperature decrease reduces the chemical exergy of the exhaust gases, and (3) the chemical exergy of the exhaust gases exhibit the same behavior as the combustion emissions since its evolution follows the mass fractions of exhaust gases species.

To further extend the applicability of the entropy generation analysis technique to other combustion modes, Dressler et al. [139] recently investigated the entropy generation in stratified premixed flames relying on a chemistry tabulation strategy in coupling with the T-FDF equation solved by means of the Eulerian stochastic field method. The approach was first validated for laminar stratified flame and then adopted to compute the Darmstadt stratified burner under two operating conditions featuring the same level of stratification but different levels of shear.

The heat transfer has the major contribution in entropy generation exceeding the chemical reaction, which represents the second main responsible for the exergy destruction. The authors also revealed the effect of shear level variation on the entropy production: higher shear leads to higher entropy generation through heat, mixing and viscous dissipation and decreases the contribution of chemical reaction to the total entropy produced, and this effect is largely noticeable for the heat transfer source term.

The reported works [32,33,138,139] (see also Table A1 in Appendix A) focused on the entropy generation analysis in combustion systems within the LES framework constitute a large step towards building efficient, accurate and inexpensive tools for second law analysis. Their evaluation shall be further performed in other combustion configurations and extended to complex or/and practical applications, such as multiphase reactive flows and IC engines.

Moreover, effects, such as non-adiabaticity, differential diffusion, phase change, particle turbulent dispersion, collision, coalescence and breakup, are not yet included. Such tasks are required to finally provide an entropy-based optimization tool that deals suitably with a wide range of combustion modes in combustion systems and includes the main involved phenomena in entropy generation process.

### 5.3. Near-Wall Challenges in EGA Using LES

In many energy systems, such as internal combustion engines, gas turbines, heat exchangers and converter monolith channels in after-treatment devices, wall-bounded processes take place and involve complex interactions that may include conjugate heat transfer. It is well-known that LES is itself challenging near the wall. This is mainly due to the thin momentum, thermal or/and concentration boundary layers at the wall surface that have to be fully resolved in classical LES and requires fine spatial resolution, in particular for turbulent flows with high Reynolds and Prandtl/Schmidt numbers.

To overcome this issue, it is common practice in LES to use a near-wall modeling approach to reduce the required computational effort of the simulation. In general, such near-wall modeling strategies can be categorized into three approaches—namely, those based on wall functions (WFLES), two-layer RANS–LES (zonal LES) and hybrid RANS–LES methods [140]. Recent reviews can be found in [141].

In [142], the authors performed first entropy generation analysis using the various models that include conjugate heat transfer. To the author’s present knowledge, published reports on EGA using LES in near-wall that consider species concentration together with chemical reactions are not yet available. This aspect requires special attention and more appropriate research activities, in which the wall properties and structure (solid, porous, rough, etc.) should be considered as they are of interest in many applications other than IC engines, gas turbines and power plants, such as fire safety.

## 6. EGA as an Optimization Tool

Exploiting the ability to quantify the degree of irreversibility by means of the entropy generation, which can be considered as a function that depends on the path followed by the thermodynamic process and on geometrical parameters of the system design, made it possible to use the Entropy Generation Analysis along with the Entropy Generation Minimization (EGM) [8,143]; see also [31] for optimization purposes.

Bejan [8] was the first to provide a review of the development and adoption of the Entropy generation minimization (finite time thermodynamics, or thermodynamic optimization) in several sectors of relevant practical importance, such as cryogenics, heat transfer, education, storage systems, solar power plants, nuclear and fossil power plants, as well as refrigerators. Ordonez et al. [143] reported, in their recent review on the thermodynamic methods of analysis, the concepts of the exergy and entropy generation in order to assess system performance by means of metrics that quantify the degrees of irreversibility.

In this respect, other processes may require Entropy Generation Maximization for their optimization [144], such as a coupled biosphere–atmosphere system or multiphase system with spray atomization process and its subsequent evaporation in combustion chambers.

As usual in optimization tasks, both methods enable the formulation of constrained optimization problems in which the objective function is the entropy generation, the degrees of freedom include typically operational and geometrical parameters, and the constraints are associated with the finite resources (heat transfer areas, system volume, etc.) [143].

The method encompasses both modeling and optimization and can be implemented in following three steps: First, a model for the system is constructed using the laws of thermodynamics as a framework. Fluxes and interactions are incorporated into the model from related disciplines, typically heat transfer and fluid mechanics. These introduce geometrical features of the system and related degrees of freedom.

Secondly, the entropy generation (or its rate) is computed generally in one of following methods. In the direct or lumped method, the second law is utilized directly to compute the total entropy generation rate in the system where it equals the net outflow of entropy from the system. In the second method, the local entropy generation rates (per unit volume) due to different phenomena (viscous friction, heat transfer, mass diffusion, coupling term between the mass and heat transfer and chemical reaction) present in the system are calculated. This tends to be used in connection with CFD approaches.

Third, the entropy generation is minimized by solving a constrained optimization problem. This can, on occasion, be accomplished analytically but often requires numerical approaches. In principle, any suitable function minimization approach can be used [143].

Arjmandi and Amani [145] simulated the turbulent combustion of a mixed bluff-body-swirl stabilized flame in a gas turbine combustion chamber. RANS-based governing equations were used (continuity, momentum, species transport and ideal gas law) together with the RNG k-ε model in conjunction with the eddy-dissipation model for the averaged reaction rate. Two methods, i.e., the direct and indirect methods, for calculating the entropy generation were applied.

The effects of different parameters, including the equivalence ratio, fuel-inlet flow rate, bluff-size ratio, swirl number and air-inlet velocity, on the entropy generation could be monitored. This allowed to perform the design process of the combustion chamber by proposing the optimum value of each parameter based on the EGM method, i.e., by minimizing the total entropy generation under the two maximum allowable temperature and size constraints.

Even though the methods, which involve different approximations, differ by about 6% in average, the entropy generation rate due to chemical reaction amounts the largest portion of the total entropy generation. The entropy generations due to chemical reaction and heat transfer are opposing factors whose trade-off results in the optimum design condition of this chamber. Other contributions can be mentioned.

Nevertheless, an overview of contributions dealing with optimization of systems systems involving mass transfer, chemical reactions and other transport phenomena can be found in [5], while further examples of entropy generation minimization and thermodynamic optimizations are provided in a recent mini-review [143].

In framework of LES, Ries et al. [31] were the first to apply the entropy generation analysis to characterize and optimize a turbulent impinging jet on a heated solid surface. Chemical reactions were not considered at this stage. Thereby, model equations that allow calculation of entropy generation rates in the post-processing phase of LES to ensure economical computation costs have been suggested and used.

The impacts of plate inclinations and Reynolds numbers on the turbulent heat and fluid flow properties were investigated numerically, and the implication on the thermodynamic performance of such flow arrangements was deduced. An inclination angle of 90 degree allowed the most efficient use of energy. By increasing the Reynolds number, the heat transfer intensifies and the second law efficiency of the system increases. Such efforts using LES shall be extended to optimize combustion systems by means of EGA.

## 7. Conclusions

This paper focused on providing an overview of different contributions dedicated to entropy generation analysis in turbulent combustion systems. We covered the effects of various parametric studies, including wall boundedness conditions, flow operating conditions, combustion regimes, fuels along with alternative fuels and application geometries.

First, the difficulties of performing comprehensive experiments that may support the understanding of entropy generation phenomena were outlined. Then, different approaches and models to evaluate the entropy generation source terms in the context of DNS, RANS and LES were reviewed. In particular, three main methodologies or modeling degrees for the treatment of EGA using RANS and LES were outlined. Secondly, the description paths were classified in terms of experimental investigations and numerical simulations in which various applications were presented and discussed. The latter include generic or canonical configurations, IC engines, gas turbines and power plants.

In terms of the criteria selected for this review, the following inferences can be drawn. First, in terms of the models and approaches for entropy generation analysis:

- Entropy generation modeling using DNS is still computationally expensive and difficult for detailed reaction mechanisms and complex reacting flow configurations.
- In the RANS context, the dissipative nature of the turbulence models as well as the inability to accurately predict the turbulence–chemistry interactions despite of the use of PDF methods, make the approaches used for entropy generation prediction not very accurate, and they can only be applied as a tool to quickly gain a general impression about the second law analysis of the investigated system.
- Investigations within the LES context emerge as promising since new approaches are introduced that are not computationally expensive while being accurate, particularly in representing turbulence–chemistry interactions.
- Different approaches suggested to quantify the entropy generations source terms in the different turbulence framework are still dealing with single phase turbulent flows. Entropy generated in turbulent multiphase flows or sprays, including phase change as well as other processes, such as breakup, collision, coalescence and turbulent dispersion, still need to be considered.
- Soret and Dufour effects are rarely included in entropy generation modeling approaches since, in most of hydrocarbon-air and hydrogen-air flames, these effects have an insignificant role.

Secondly, in terms of application complexity in both experimental and numerical modeling aspects:

- Most experimental investigations are performed in IC engines; however, the tendency is increasing in gas turbines and power plants. All these investigations are focused on simply calculating the exergy efficiency through an exergy balance of the system and observing the effects of certain operating parameters on the gained exergy.
- Experiments found in the literature demonstrated the limitations of experiment-based investigations, since the different entropy generation source terms during combustion and the contribution of each term cannot yet be retrieved by the existing measurement techniques.
- Numerical applications of entropy generation analysis using DNS are rarely found in the literature. Despite the fact that the existing simulations are performed using a single step reaction mechanism, which surely affected the accuracy of the quantitative prediction of the entropy generation analysis, the provided findings remain qualitatively valid, such as the great influence of the wall boundary conditions on entropy generation.
- RANS-based simulations as performed in the literature to quantify the entropy generation source terms allow the report of the major contributions of heat transfer and chemical reactions and to outline the effects of certain operating conditions on the exergy efficiency in IC engines. The presented applications were limited to simply use the exergy balance for computing the exergy efficiency without giving access to the different entropy source terms especially during combustion. Nevertheless, they made it possible to gain some insights into the relationship between the soot formation and entropy production. Such RANS-based investigations were performed under several assumptions for the sake of simplicity, which can affect the accuracy of the results.
- Different approaches for entropy generation analysis as suggested in the LES context were tested only in Sandia and stratified flames. In both cases, the heat transfer is primarily responsible for entropy generation followed by the chemical reaction.
- The assessment of the new developed approaches shall be further performed in other combustion configurations and extended to complex or/and practical applications, such as multiphase reactive flows and IC engines. Moreover, effects, including non-adiabaticity, differential diffusion, phase change, particle turbulent dispersion, collision, coalescence and breakup, shall be integrated in order to result in an efficient, accurate and inexpensive tool for second law analysis.

The listed remaining tasks are required to finally provide an entropy-based optimization tool that deals with a suitably wide range of combustion modes in combustion systems and includes the main involved phenomena in the entropy generation process.

## Figures and Tables

**Table 1:** Integral values of entropy generation rates and entropy flux for turbulent flame obtained by using RNG k-ϵ model (Reynolds-averaging-based approach) [111].

${\bar{\Pi }}_{v}^{M}$ (W/K)	${\bar{\Pi }}_{v}^{t}$ (W/K)	${\bar{\Pi }}_{M}^{q}$ (W/K)	${\bar{\Pi }}_{t}^{q}$ (W/K)	${\bar{\Pi }}_{M}^{d}$ (W/K)	${\bar{\Pi }}_{t}^{d}$ (W/K)	${\bar{\Pi }}_{ch}$ (W/K)	${\bar{\Pi }}_{gen}$ (W/K)	Entropy Flux (W/K)	Error (%)
8.1 × $10^{-6}$	0.0011	0.456	28.499	0.0283	2.896	41.967	73.847	69.397	6.4

## Data Availability

Not applicable.

## Nomenclature

**List of symbols**		**List of greek letter**	
*Ex*	Exergy	Ω	Angular speed
*T*	Temperature	Φ	Equivalence ratio
*C_p_*	Specific heat capacity	ρ	Density
*LHV*	Lower heat value	λ	Thermal conductivity
*Tr*	Engine torque	τ	Viscous stress
*P*	Pressure	μ	Dynamic viscosity
*V*	Volume	ν	Stoichiometric coefficients
*Q*	Heat flux rate	β	Dimensionless number (=1)
*m*	Mass flow	α	Specie
*R*	Gas constant	Π	Entropy source term
*Y*	Mass fraction	ω	Reaction source term
*u*	Velocity	ϕ	flow variable
*t*	time	ε	Dissipation rate
*F*	Body forces	ψ	Mass fraction variance
*e*	Energy	θ	Temperature variance
*h*	Specific enthalpy	χ	Scalar dissipation
*J*	Diffusive mass flux	γ	Ratio of specific heats
*Ns*	Number of species	φ	Quantity of unresolvedness
*M*	Molecular mass	Ψ	Irreversibility ratio
*r*	Molar net rate production	υ	Kinematic viscosity
*I*	Identity tensor	δ	Kronecker delta
*S*	Source term	Δ	Grid filter width
*c*	Molar concentration	Θ	Crank angle
*K*	Rate constant of chemical reaction	**List of subscripts**	
*Ea*	Activation energy	*in*	Input
*A*	Arrhenius pre-exponential	*dest*	Destruction
*s*	entropy	0	Ambiant
*D*	Diffusion coefficient	*ch*	Chemical
*Le*	Lewis number	*ph*	Physical
*X*	Mole fraction	*gen*	Generation
*x*	spatial coordinate	*r*	Reaction
*k*	Turbulent kinetic energy	*m*	Mixture
*Z*	Mixture fraction	*i*, *j*	Coordinate directions
*Ma*	Mach number	*q*	Heat
$S_{L}$	Laminar burning velocity	vs.	Viscous
*f*	Probability density function	*d*	Diffusion
*Sc*	Schmidt number	*L*	Local
*g*	free Gibbs energy	*c*	Progress variable
*Pr*	Prandtl number	*t*	Turbulent
**List of superscripts**		*sgs*	Sub-grid scale
res	Resolved

## Appendix A

**Table A1:** A summary of previous works on entropy generation in various applications using different numerical (modeling) and experimental approaches.

Application	Components with Reacting Flow	1D	RANS	LES	DNS	Entropy Source Terms Calculation Method	Exp.
Power plant	Combustion Chamber	[57,58,59,60,61,63,65,67,68]	-	-	-	Skeletal	[57,58,68]
Stationary gas turbine	Combustion Chamber	[71,72,73]	-	-	-	Skeletal	-
Aircraft gas turbine	Combustion Chamber	[74,75,76,77,78,79]	-	-	-	Skeletal	-
IC engine	Combustion Chamber	-	[118,119,120,121,122]	-	-	Skeletal	[37,38,39,40,41,42,43,44,45,46]
Jet flame	non premixed	-	[21,22,111,113,115]	[32,33,138]	-	PDF/tabulated/turbulence/DNS data/thermodynamic	-
	premixed	[109]	-	[139]	[20,27,108]	tabulated/turbulence/DNS data	-
	Dilution H2	-	[21]	-	-	turbulence	-
